# Supplementary material for: Are carbon emissions trading and green financial instruments synergistic? -Comprehensive quantitative research based on content analysis
Source: PLoS One. 2024 Mar 7;19(3):e0298601. doi: 10.1371/journal.pone.0298601 (PMC10919601; doi:10.1371/journal.pone.0298601)
Supplement: S1 Data — (ZIP) [file pone.0298601.s001.zip › Data/地级市Z_F工作报告.zip!/┴╔─■/│»╤⌠╩╨/└┤╫╘╣1⁄2╓┌║┼í╛┬φ┐╦╩2╛▌═°í┐_2018.docx]

各位代表：

　　现在，我代表市人民政府向大会报告工作，请予审议，并请各位政协委员和其他列席人员提出意见。

2017年及本届政府工作回顾

　　2017年，在省委、省政府的亲切关怀和市委的坚强领导下，在市人大及其常委会和市政协的监督支持下，我们团结带领全市各族人民，统筹推进“五位一体”总体布局、协调推进“四个全面”战略布局，抢抓机遇，攻坚克难，实现了经济筑底企稳、民生不断改善、社会和谐稳定。

　　预计全年完成地区生产总值765亿元，增长4%；公共财政预算收入57.9亿元，增长13.5%；固定资产投资252亿元，增长3.3%；社会消费品零售总额458.2亿元，增长2.1%；城乡常住居民人均可支配收入分别达到23948元和11865元，分别增长7%和6%。主要经济指标增速由负转正，部分指标超额完成预期目标。一年来，我们主要做了以下工作：

　　一、推动经济稳定增长

　　强化“五个一工程”的引领和载体作用，分类出台支持政策，统筹推进体制机制创新、基础设施建设、融资平台搭建。建设标准化厂房434万平方米，入驻企业399家。朝阳县新能源汽车及汽车零部件、建平保温材料、喀左紫陶建材等产业集群加快建设，北票施可丰复合肥、凌源航天凌河整车等项目扎实推进，双塔环城生态旅游带初具规模，龙城黑猫炭黑项目建成投产。

　　把扩大有效投资摆在经济工作首位，做实“开复工项目库”“招商项目储备库”，采取抓项目储备、要素保障、投资进度和涉企服务等措施，开工建设一批稳增长、调结构、补短板项目。全年实施亿元以上建设项目192个、10亿元以上建设项目9个。

　　瞄准京津冀和南方地区产业转移，开展大型招商活动23次，引进内资160亿元，利用外资6000万美元，新落地项目152个。加强政府与社会资本合作，完成PPP储备项目100个，33个项目进入省重点项目库。主动对接“一带一路”建设，进出口总额实现40.5亿元。

　　认真贯彻中央新一轮东北振兴和省委、省政府新一轮突破辽西北、县域经济发展战略部署，制定相关规划计划，争取国家和省支持资金188亿元，振兴发展政策举措得到有效落实。

　　二、推动产业结构转型升级

　　扎实推进农产品加工园区建设，龙头企业达362家。新增节水灌溉面积20.7万亩，实现设施农业产值100亿元，建立土地股份合作社610家。调减玉米种植面积11.6万亩。面对春季持续干旱，通过加强大田生产服务、开展人工增雨等措施，粮食生产喜获丰收，产量达到51.6亿斤。

　　开展服务存量企业行动，支持凌钢、浪马轮胎、朝柴等重点企业发展，帮助中小企业渡过难关，扭转了工业经济增长乏力的被动局面。新增规上企业53户。实现规模以上工业增加值87亿元，增长8%。

　　推动传统服务业转型升级，加强商品物流体系建设，发展壮大电子商务、会展等新兴业态。强化重点景区对外合资合作，文化旅游硬件条件逐渐改善。发挥金融服务实体经济作用，积极防控金融风险，新增融资180亿元。

　　三、推动改革创新不断深化

　　持续推进供给侧结构性改革，化解煤炭产能85万吨，9万吨以下小煤矿全部关闭。关闭“地条钢”企业1家，淘汰违规落后小高炉6座。完成商品房销售面积118万平方米。推进9家重点企业债转股。减轻企业和群众负担1.1亿元。

　　继续深化“放管服”改革，取消调整行政职权168项，行政审批承诺办理时限压缩80%以上，审批前置要件削减70%以上。全面实行“双随机、一公开”监管，探索推行“多证合一、一照一码”“证照联办、一照通”等商事制度改革。完成生产经营类事业单位改革和经济开发区“多规合一”改革。

　　积极开展“营商环境建设年”活动，成立营商环境建设监督管理局，制定出台系列制度措施，强力开展有针对性的专项整治，着力解决政府诚信低、政策落地难等突出问题，营商环境得到明显改善。

　　深入推进国资国企改革，城发、水务、文旅、国恒集团组建完成并运行，凌钢营业收入首次超过200亿元，朝阳银行资产总额突破800亿元、主要经营指标三年翻一番。市属国企公司制改革全面完成，混合所有制改革起步良好，经营效益进一步提高。

　　实施科技创新“五大工程”，完成科技成果转化50项，新增高新技术企业19家。深入开展大众创业、万众创新活动，创业孵化基地达7.8万平方米，新增市场主体2.3万户。

　　四、推动城乡建设取得新成效

　　启动国家森林城市创建工作，完成植树造林面积83.9万亩、水土流失综合治理面积124万亩。全面推行“河长制”，凌河流域水质进一步改善。淘汰黄标车和老旧车辆、拆除小锅炉、禁烧秸秆等工作扎实推进，空气优良天数达到280天。中央环保督察组交办信访案件全部结案。我市获批全国第三批低碳城市试点、国家首批气候适应型城市建设试点。

　　修编城市总体规划，编制旅游等系列专项规划。友谊大街全线贯通，环城生态绿廊等重点工程积极推进。国电热电联产项目建成投产，城区北部集中供热工程加快实施，新增集中供热面积400万平方米。拓宽改造城市道路14公里，新建改建农村公路1194公里。建设美丽乡村38个。开展市容环境集中整治，车辆占道停放、乱搭乱建等群众反映强烈的问题得到初步解决。

　　抢抓京沈客专开通带来的重大机遇，坚持顶层设计、全域联动、规划引领，明确产业方向，强化基础设施，谋划迎接高铁时代工作扎实有效推进。

　　五、推动民生福祉持续改善

　　强力推进脱贫攻坚，狠抓产业、金融、健康、教育、企业、低保兜底等重点扶贫措施，瞄准贫困群众精准帮扶，激发贫困人口内生动力，加强监督考核，完成193个贫困村销号、5.35万贫困人口脱贫任务。

全面推进就业和社保等工作。城镇新增就业1.6万人，登记失业率控制在3.9%，朝阳县、龙城区获批国家支持农民工等人员返乡创业试点地区。企业养老金、城乡低保金标准稳步提高，并按时足额发放。开工棚户区改造工程1.29万套，货币化安置率达到100%。建设农村饮水安全工程38处，实施农村危房改造8392户，完成老旧住宅小区改造50万平方米。

持续加强公共服务供给能力建设。协调发展各级各类教育，新建改扩建乡镇标准化中心幼儿园10所，实施“全面改薄”项目学校125所，喀左县、龙城区通过“义务教育发展基本均衡县”国家认定，高中教育质量明显提升，职业教育活力进一步增强，师专转型升级取得重要进展。实施健康朝阳战略，不断提升公共卫生服务水平，全面推开公立医院综合改革，8家城市公立医院全部取消药品加成，市第二医院晋升三甲医院，全市人均期望寿命达到76.5岁。加强食品药品安全监管，食品药品满意度不断提高。推进文化惠民工程，新建农村文化活动广场33处，群众文化艺术节成为国家第三批公共文化服务体系示范项目。广泛开展全民健身活动，新建农民健身工程130处。

　　积极推进社会治理创新，立体化、信息化治安防控体系不断完善，应急管理能力进一步增强，安全生产和信访形势总体稳定。圆满完成十九大维稳安保任务，创造全省最好成绩。有效应对“8.3”特大暴雨，实现大雨大汛零伤亡。民族宗教、司法行政工作全面加强，国防动员、人防和民兵预备役工作呈现新局面。住房公积金、邮政、档案、援疆、外事侨务及老年人、妇女儿童、残疾人权益保障等工作取得新成绩。

2017年是本届政府的收官之年。过去五年，我们在振兴发展道路上奋勇前进，在朝阳历史上书写了新的篇章！

　　五年来，我们坚持以经济建设为中心，发展动能不断集聚。圆满完成“十二五”规划目标任务，“十三五”规划顺利实施。全力开展“五个一工程”建设，7个县（市）区实现省级经济开发区、服务业集聚区全覆盖，2个农业园区成为省级示范园区，1个新市镇荣获国家卫生镇称号，7个乡镇进入省级特色乡镇培育名单，5个景区晋升4A级景区，培育了新的发展动能，形成了新的发展空间。持续开展项目年活动，中航科技、中信、万达等一批大企业大集团落地朝阳，发展后劲进一步增强。	（下转第二版）

　　五年来，我们坚持以结构调整为重点，产业基础进一步夯实。现代农业迈出新步伐，产业化、规模化和设施化水平持续提升，设施农业、杂粮、畜牧业、林果业等优势产业取得长足发展，农业共营制改革全面推进，农产品区域公用品牌创建迈出重要步伐。工业结构调整不断深入，中国制造2025朝阳行动纲要全面实施，冶金、汽车及零部件等传统产业逐步调优，新能源汽车、新能源及高端装备、金属新材料等新兴产业加速成长，高新技术企业数量比上个五年增长88%。服务业提速发展，集聚区建设稳步推进，旅游业总收入年均增长13%，引进金融机构18家，4户企业在“新三板”挂牌，基金设立实现“零”的突破，现代服务业占比由五年前的17%提升到25%。

　　五年来，我们坚持以改革开放为动力，经济社会发展活力持续增强。成功纳入国家行政审批制度改革试点市，在全省率先组建行政审批局和市场监管局，实现一枚公章管审批、一个部门管市场。园（景）区、事业单位、商事制度、财税、金融、投融资等领域改革扎实推进。突出质量管理，浪马轮胎获“省长质量奖银奖”，实现了“省长质量奖”零的突破。新创中国驰名商标3件、辽宁省著名商标38件。坚持不懈推进对外开放，引进内资、利用外资、出口创汇、实施亿元以上项目数量分别是上个五年的1.4倍、1.1倍、1.7倍和2倍。扎实开展大众创业、万众创新活动，龙城区创业创新孵化基地被评为全国示范基地。

　　五年来，我们坚持以完善城乡环境为支撑，发展保障能力持续提升。投入基础设施建设和环境改造资金39亿元，完成造林绿化面积521万亩，凌河整治、大气治理、人工影响天气等工作取得重要进展，我市成功晋升全国绿化模范城、省级环保模范城。城乡道路交通网络逐步完善，电网规模进一步扩大，涉农行政村亮化实现全覆盖。新型城镇化步伐不断加快，城镇化率提高到45.3%。京沈客专、喀赤客专、朝凌客专和锦承、叶赤铁路扩能改造等项目加快建设，朝阳至北京、上海、天津、大连、松原等航线陆续开通，外环路、燕白滨河路、文明路建成并投入使用。

　　五年来，我们坚持以提高人民生活水平为根本，社会大局保持和谐稳定。累计投入民生事业资金749亿元，实施一大批惠及城乡群众的民生工程。社会消费品零售总额、城乡常住居民人均可支配收入年均分别增长7.9%、8%和9%。脱贫攻坚取得突破性进展，每个县（市）区都实施2个以上产业扶贫项目，实现315个贫困村销号、10.6万贫困人口脱贫，贫困发生率从6.9%下降到2.5%。社会保障体系逐步完善，城乡低保和企业退休人员养老金分别增长45.7%、71.4%和40.4%。城镇居民医保和新农合政府补助标准分别提高60.7%和87.5%。积极打造“十分钟便民服务圈”，社区便民服务体系初步形成。完成棚户区改造5.4万户、暖房子工程80万平方米。社会事业全面进步，平安朝阳、法治朝阳建设深入开展，人民群众获得感、幸福感、安全感更加充实、更有保障、更可持续。

　　五年来，我们坚持以加强自身建设为保障，政府执行力和公信力不断增强。落实全面从严治党要求，深入开展党的群众路线教育实践活动、“讲诚信、懂规矩、守纪律”主题教育和“三严三实”专题教育，推进“两学一做”学习教育常态化制度化，全力修复政治生态，政府系统干部作风持续转变。坚持依法行政，推行政务公开，加强风险管控，严格执行重大事项集体决策制度。认真落实党风廉政建设责任制，加强行政监察、审计监督、统计监督和督查督办，政府廉政建设取得新成效。坚决执行中央“八项规定”，公务接待费用年均下降27.3%，清理腾退办公用房11万平方米。五年共办理人大代表议案和建议349件、政协提案967件，满意率达99%以上。

　　过去五年，七个县（市）区开拓进取、砥砺前行，经济社会发展都取得了新的成就：

　　北票市环保装备产业成为新一轮突破辽西北战略重点推动产业，汽摩配产业集群初具规模。大黑山景区晋升4A级景区，天鹅湖景区列入国家发改委PPP示范项目。“1+4”扶贫模式受到国务院第三次大督查通报表扬。 

　　凌源市以汽车及零部件、装备制造和新型复合材料为主导的产业格局初步形成，汽车产业稳步发展，石墨烯产业园项目实现成果转化并上市推广。设施农业优势进一步扩大，获批国家现代农业示范区。

　　朝阳县产业结构调整取得重要进展，开发区标准化厂房、农产品物流园、柳城商贸城、三燕古城等一批重大项目全面实施。荣获“全国农产品质量安全县”称号，建立全市首个军民融合创新示范基地。

　　建平县城区“一环五横八纵”路网和“一河三带九园”生态景观体系初步形成。家居和新型建材产业园建设快速推进。成功引进温氏、牧原、正邦等企业，正在朝着东北地区最大全产业链生猪养殖基地迈进。 喀左县装备制造产业园转型升级取得突破性进展。龙凤山、浴龙谷、龙源3个旅游景区晋升4A级景区。成功创建国家级出口蔬菜质量安全示范区和农业可持续发展试验示范区。荣获全国综治最高荣誉“长安杯”。

　　双塔区经济技术开发区迁址工作全面完成，基础设施水平显著提升。服务业增加值突破100亿元，成为全省服务业发展先进城区。万达、新华、文化、珠江等四大商圈初步形成点式集聚、错位发展格局。

　　龙城区财政收入连续两年保持20%以上的增速。加华电子、光达化工等一批老企业在合资合作和技术改造中焕发生机。成功创建国家级出口农产品质量安全示范区，燕山湖庙子沟被评为全国运动休闲特色小镇。

　　各位代表，过去五年是新世纪以来朝阳发展最困难、经受的考验最多、遇到的矛盾最突出的时期。我们正确研判形势，直面问题和挑战，始终坚持党的领导，切实增强“四个意识”，坚决同以习近平同志为核心的党中央保持高度一致，认真贯彻落实省委、省政府和市委的决策部署，牢牢把握正确的政治方向；我们坚持稳中求进总基调，牢固树立新发展理念，不断适应把握引领新常态，在发展中求转变，在转变中谋发展；我们努力破除思维定势、工作惯性和路径依赖，向改革要动力，向创新要活力，向开放要空间，以抓铁有痕、踏石留印的作风，确保各项工作落地生根；我们坚持远近结合、统筹推进，既注重解决当前经济运行的突出矛盾，又科学谋划事关长远发展的重大事项，既重视改善硬环境，又大力提升软环境，既注重推动发展，又狠抓风险防控，走出了一条符合朝阳实际的振兴发展之路；我们自觉践行以人民为中心的发展理念，持续增加民生投入，切实提高公共服务能力，使发展成果更多更公平惠及全市人民。

　　各位代表，过去五年取得的成绩是省委、省政府高度重视、亲切关怀的结果；是市委正确领导、科学民主决策的结果；是市人大、市政协有效监督、大力支持的结果；是全市上下同心同德、团结奋斗的结果。在此，我代表市人民政府，向全市各族人民，向人大代表、政协委员，向各民主党派、工商联、无党派人士和各人民团体，向驻朝中省直单位、解放军指战员和武警官兵，向所有关心支持朝阳改革发展的社会各界人士，表示崇高的敬意和衷心的感谢！ 

　　总结五年工作，我们清醒地认识到还存在一些亟待解决的困难和问题。一是发展不平衡不充分。经济总量小，发展质量和效益不高，实体经济发展不快，城乡二元结构没有得到根本改变。二是发展动能不足。对外开放度低，引进落地大项目和好项目少，新旧动能接续转换需要较长一段过程，经济持续稳定向好的基础尚不牢固。三是结构调整步伐不快。供给侧结构性改革不到位，传统产业转型升级进展缓慢，新兴产业成长不足，科技创新带动作用没有有效释放。四是体制机制不优。国资国企改革深度不够，民营经济发展不足，创新创业氛围不浓，良好的政商关系尚未完全形成，经济发展软环境有待进一步改善。五是民生和社会建设亟待加强。生态环境依然脆弱，精准扶贫存在薄弱环节，公共服务能力不足，影响发展的潜在风险和隐患仍然较多，就业、教育、医疗、居住、养老等群众关心的热点难点问题没有得到有效解决。六是政府职能转变不到位。解放思想的程度、狠抓落实的力度还有差距，少数干部责任意识和执行力不强，工作效率不高，不敢担当、不愿作为、不善作为等问题仍很突出，管理和服务水平有待进一步提高。我们一定高度重视并下更大力气解决这些问题，竭尽全力做好政府工作，绝不辜负全市人民的期望和重托。

　　未来五年政府工作奋斗目标和展望

　　未来一个时期，朝阳面临推进振兴发展和进一步净化政治生态的双重任务。面对新旧矛盾叠加、风险隐患交织的挑战，我们必须做好滚石上山、爬坡过坎的准备并付诸行动。同时要认识到，党的十九大描绘了决胜全面建成小康社会、夺取新时代中国特色社会主义伟大胜利的宏伟蓝图，为在新的起点上推动朝阳经济社会发展指明了方向。当前朝阳正处于后发优势的“释放期”、多重机遇的“叠加期”、动能转换的“积蓄期”和振兴发展的“关键期”。只要我们保持定力，持续发力，抢抓机遇，积聚释放积极因素，就一定能够行稳致远，迎来更加美好的明天。

　　今后五年，我们要全面完成“十三五”规划任务，实现“十四五”良好开局，确保到2020年全面建成小康社会。在此基础上，按照党的十九大提出的“两个阶段”战略安排，进一步推进经济、政治、文化、社会和生态文明建设，全市经济实力迈上新台阶，结构调整实现新突破，改革创新达到新高度，城乡建设展现新面貌，惠民富民取得新进步，实现全面振兴发展，开启现代化建设新征程。

　　——继续深化供给侧结构性改革，在厚植产业基础中实现高质量发展。把高质量发展作为解决朝阳所有问题的关键，主要指标增速持续高于全省平均水平，重点工作走在辽西北地区前列，经济规模超越千亿元，财政收入力争突破百亿元。以“五个一工程”为载体，深入推进结构调整，推动产业向中高端迈进。坚持增量存量并重，引导冶金、汽车及零部件、建材、装备制造等传统产业改造升级，加快发展新能源汽车、新能源及高端装备、新材料、节能环保、电子信息等新兴产业。积极发展现代农业，建成全国重要的绿色农产品生产加工和物流基地。在改造提升传统服务业的同时，发展壮大物流、电子商务、健康养老等现代服务业。大力发展旅游业，引进建设一批具有牵动作用的重大项目，旅游业增加值占地区生产总值比重达到5%以上。坚持去产能、去库存、去杠杆、降成本、补短板，不断优化存量资源配置，扩大优质增量供给。 ——继续推进改革开放和科技创新，努力培育壮大发展动能。扎实推进以全国行政审批制度改革试点市建设为牵动的“放管服”改革，不断提高行政服务质量和效率，以更大的决心和勇气持续突破重点难点问题，打造更加优越的营商环境。进一步提升园（景）区体制机制创新水平，建设朝阳改革创新示范区。全面深化国资国企改革，大力支持非公有制经济发展。实施更加主动的开放战略，加快建设京津冀产业承接基地、优质农产品供应基地、清洁能源供应基地和旅游休闲养生基地。引导支持科技要素和创新资源向企业聚集，重点在冶金铸造、新能源汽车、新材料、节能环保等领域突破一批关键核心技术。高新技术企业达到100家以上，高新技术产品增加值占工业增加值比重达到50%以上。

　　——继续加大城乡建设力度，切实促进区域协调发展。深入实施乡村振兴战略和县域经济发展战略，五县（市）综合实力进一步增强，在全省位次前移，形成全新的发展格局和态势。做大做强城区经济，两区在产业发展、生态环境建设、城市建设管理、民生改善、社会治理等领域实现全面创新。加快推进城乡基础设施建设，推动京沈客专实现运营，城乡电网改造、绥凌高速公路等工程实现重要进展。培育省级特色乡镇10个，力争创建国家特色小镇2个，城镇化率达到50%以上，城乡形象全面提升。

　　——继续推进生态文明建设，打造美丽朝阳。把生态文明建设作为最基本和最基础的建设，逐步将其打造成朝阳的核心竞争力。实行最严格的环境保护制度，加大生态修复和保护力度，加快形成绿色生产生活方式，不断提升资源利用效率和生态环境质量。打好污染防治攻坚战，主要污染物排放总量持续下降，空气优良天数保持在80%以上。以创建国家森林城市为牵动，持续加大造林绿化力度，森林覆盖率达到50%以上，成为京津冀地区的重要生态屏障。继续实施碧水、蓝天、沃土工程，全面完成城区集中供热改造，加强矿山矿业综合整治、海绵城市、环城生态绿廊等重点工程建设，使天蓝、地绿、水净、城美、空气清新成为常态。

　　——继续提高民生保障水平，加强和创新社会治理。全面落实以人民为中心的发展理念，着力解决群众最关心最直接最现实的利益问题。坚持精准扶贫、精准脱贫，按照不降低标准、不吊高胃口的要求，确保到2020年5个贫困县全部摘帽、662个贫困村全部销号、17.16万贫困人口全部脱贫。实现比较充分就业，城镇登记失业率年均控制在4.5%以内。城乡居民收入增速高于经济发展速度，收入差距不断缩小。社会保障制度更加公平、更可持续，实现人人享有基本社会保障。教育、医疗卫生、体育、住房、养老等公共服务体系更加健全，基本公共服务均等化水平稳步提高。推动文化大发展大繁荣，争创国家历史文化名城。持续抓好安全生产，加强预防和化解社会矛盾机制建设，维护社会和谐稳定。做好重点民生工程，人民群众获得感、幸福感、满意感全面提升。

　　各位代表，朝阳已经进入改革发展的新时代。未来五年，我们一定在市委的领导下，紧紧依靠广大人民群众，集中各方面力量和智慧，以强烈的责任感、紧迫感、使命感，抢抓机遇，实干苦干，善做善成，毫不懈怠，向全市人民交上一份满意的答卷！

　　2018年工作安排

　　2018年是贯彻党的十九大精神的开局之年，是决胜全面建成小康社会、实施“十三五”规划承上启下的关键一年，也是新一届政府的起步之年。做好今年的政府工作，要全面贯彻党的十九大精神，以习近平新时代中国特色社会主义思想为指导，认真落实新发展理念、“四个着力”“三个推进”要求，按照省委十二届六次全会和市委十届六次全会的部署，抢抓新一轮东北老工业基地振兴、突破辽西北和县域经济发展等战略机遇，坚持稳中求进工作总基调，以供给侧结构性改革为主线，以提高发展质量和效益为中心，以“五个一工程”为龙头，统筹推进稳增长、促改革、调结构、惠民生、防风险各项工作，促进经济社会持续健康发展。

　　主要预期目标是：地区生产总值增长7%，公共财政预算收入增长6%，固定资产投资增长10%，社会消费品零售总额增长5.5%，城乡常住居民人均可支配收入分别增长7%和8%。城镇登记失业率控制在4.5%以内。

　　确定这样的目标，综合考虑了宏观经济环境和自身基础条件、全面建成小康社会进程和省委、省政府各项三年攻坚计划要求。朝阳是欠发达地区，只有在确保发展质量的同时提升发展速度，加快经济总量扩充，才能缩小与先进地区的差距。我们要进一步增强责任感和紧迫感，对标先进，自我加压，立足新起点，把握新机遇，推动振兴发展实现新跨越。

　　一、扎实推进供给侧结构性改革，推动产业向中高端迈进

　　改变我市一产大而不强、二产结构单一、三产发展不充分的现状，必须坚持壮大总量和优化结构并进、传统产业和战略新兴产业并举，切实提高发展质量，培育发展动能。

　　加快推进农业农村现代化。科学制定乡村振兴战略规划，推进农业农村逐步实现产业兴旺、生态宜居、乡风文明、治理有效、生活富裕。建立健全城乡融合发展体制机制，清除阻碍要素下乡各种障碍。进一步优化农业结构，积极发展优质高效农业，加大废弃棚改造恢复利用力度，新增设施农业3.2万亩，推动畜牧业和林果业实现新发展。全面落实粮食安全省长责任制，切实增强粮食可持续生产能力。大力推进农业产业化经营，确保1—2个农产品加工园区晋升为省级园区，市级以上重点龙头企业达到280家以上。新增节水灌溉面积13.5万亩，做好人工影响天气工作，扩大农业保险面积。进一步提高农业机械化水平，积极推广农机深松整地技术，推进农业可持续发展。以共营制改革和农村集体产权制度改革为牵动，加快推进农村承包地“三权”分置，推进土地规模化集约化经营，土地股份合作社达到990家。加快农产品现代流通体系和公用品牌建设，继续加强农产品质量安全监管，启动农产品质量安全示范市创建工作。促进农村三次产业融合发展，拓宽农民增收渠道，壮大集体经济。加强农村“三资”管理。培养造就一支懂农业、爱农村、爱农民的“三农”工作队伍。

　　促进工业转型升级。实施工业强市战略，落实中国制造2025朝阳行动纲要，改造升级冶金、汽车及零部件、建材和装备制造等传统产业，积极推进凌钢高品质优特钢研发、凌源汽车生产、浪马轮胎技改、矿业资源精深加工等项目建设，帮助和支持中小企业转型发展。推动朝重等城区老工业企业搬迁改造。加快环保装备、金属新材料、包装新材料、新能源、新能源汽车和电器等新兴产业发展，形成多点支撑、特色鲜明、竞争力强的新型工业化格局。每个经济开发区都要打造1—2个销售收入超过百亿元的产业集群。规模以上工业增加值增长7%。

　　加快发展服务业。依托7个省级重点服务业集聚区，全力引进建设一批大项目、好项目，提高生产性服务业专业化、生活性服务业精细化水平。在抓好传统服务业提质升级的同时，进一步发展壮大物流、电子商务、健康养老、服务外包、文化体育产业等现代服务业，电子商务交易额增长10%以上。引进和新建金融机构，发展多层次资本市场，完成融资140亿元，提高金融服务实体经济水平。

　　 积极发展文化旅游业。加快景区功能提升，深化景区对外合资合作，启动建设三燕古文化街区、天鹅湖景区、热水汤开发、中青旅文化旅游小镇、三燕冰雪小镇、华录乐动旅游小镇等一批文化旅游项目。完善旅游“一卡通”年票制度，继续实施“引客入朝工程”，与周边地区开展广泛、多层次的区域旅游合作。喀左、凌源、北票创建国家全域旅游示范区。

　　落实“三去一降一补”任务。巩固化解煤炭、钢铁过剩产能成果，坚决防止“地条钢”死灰复燃。加快建立多主体供应、多渠道保障、租购并举的住房制度，进一步缩短去化周期，完成省政府下达的棚户区改造任务，促进房地产市场平稳健康发展，商品房销售面积增长5%。降低企业税费负担和交易成本。补齐发展短板，提高经济社会协调和可持续发展能力。

　　二、实施项目牵动战略，支撑经济持续向好发展 牢固树立“抓项目就是抓发展、谋项目就是谋未来”的理念，盘活存量，吸引增量，扩大总量。

　　积极推进“五个一工程”建设。推行“大部门制”“扁平化”管理，探索实行园区管理公司化、园区建设市场化，深化“多规合一”改革。拓宽融资渠道，完善基础设施，新建标准化厂房260万平方米以上，进一步提升承载能力。明晰产业定位，狠抓项目引进和建设，加快培育壮大重点产业集群。每个县（市）分别规划建设一个2—3平方公里的工业扶贫产业园。积极推进市经济技术开发区、高新技术产业园区和北票经济开发区晋升国家级开发区工作。

　　全力抓好项目年建设。充实“年度招商项目库”，做实“年度开复工项目库”，细化实化要素保障、重点项目全程代办、招商项目考核等工作措施。积极推进北票汽摩配产业园、凌源石墨烯新材料产业园、朝阳县电梯产业园、建平家居产业园、喀左装备制造产业园、双塔金属新材料产业园、龙城新材料包装产业园建设，加快推进迎接高铁时代相关重点项目。继续谋划实施一批PPP项目，在破解资金瓶颈、推进基础设施和公益事业、化解政府债务等方面实现多赢。精选一批重点项目，落实市县两级领导和部门包保责任。实施亿元以上重点项目260个，每个产业园区都要新建一个10亿元以上的核心项目。

　　狠抓对外开放。依托资源和产业优势，务实高效开展京津冀和南方地区产业转移招商，强力推进以商招商、专业招商、定点招商和集群招商。实施市级领导牵头招商，进一步落实县（市）区和园（景）区招商引资主体责任，明确部门招商引资任务职责，建立健全跟踪调度和督查考核机制，确保引进域外资金增长8%、实际利用外资增长6%。积极开展镇江对口合作和大连对口帮扶活动，加快产企对接，探索“飞地经济”“异地园区”。推进优势资源和富余产能走出去，扩大对“一带一路”沿线国家和地区的开放合作，进出口总额均增长5%。

　　实施存量企业改造提升工程。继续开展服务存量企业专项行动，建立市领导帮扶企业制度，举办地工产品产销衔接会，搭建银企对接平台，着力帮助现有企业特别是停产半停产企业解决实际问题，妥善处置“僵尸企业”。加快凌钢、鞍钢朝阳公司、朝柴、航天长峰电源、利浩型材、飞马铸造等骨干企业改造升级，促进佛瑞达、天翼国基、金麟铁精粉、光达化工等企业加速成长。培育优秀企业家队伍，提升企业经营管理水平。

　　三、持续深化改革，不断释放发展活力

　　以庆祝改革开放四十周年为契机，积极推进深化改革年活动，在重点领域和关键环节改革上取得新突破，为高质量发展提供体制机制保障。

　　打造一流投资营商环境。深化“放管服”改革，推行“多证合一、多评合一、多图联审”和网上审批，进一步做好“双随机、一公开”工作。坚持标本兼治，持续开展专项行动，强力整治企业和群众反映突出的问题，打造更加优越的区域发展软环境。

　　深化行政管理体制改革。推进环境监察体制、城市建设管理体制改革，抓好县、乡财政体制改革，进一步深化事业单位改革、供销社综合改革。继续加大向两区放权力度，推进城区经济发展。

　　强力推进国资国企改革。推动现有国有企业集团发展壮大，加快组建新的国企集团和国有资本运营公司，建立产权清晰、权责明确、政企分开、管理科学的现代企业制度。进一步健全国资监管体系，确保国有资产保值增值。积极推进股权多元化改革，发展混合所有制经济。

　　大力支持民营经济发展。营造良好的政策、创新和市场环境，为民营经济发展松绑减负，提供周到服务。实施市场准入负面清单制度，健全融资、担保等平台，支持中小微民营企业发展壮大。

　　加快推动创新发展。加强与科研院所、大专院校合作，进一步完善产学研协同创新机制，实施科技成果转化项目50个以上。加快技术、产品、业态创新，新增高新技术企业20家以上。以“五个一工程”为依托，培育创新人才队伍，拓宽人才引进渠道。积极推进大众创业、万众创新，新增市场主体2.6万户。实施质量、品牌战略，加快智慧城市建设。

　　切实做好防风险工作。加强企业养老保险基金收支管理，多渠道筹集资金，确保退休人员养老金按时足额发放。将政府债务风险防范化解作为硬性指标纳入对各县（市）区考核内容，在严控增量的同时，有效化解存量。高度重视金融风险防范，促进金融与实体经济、房地产等的良性循环，坚决打击违法违规金融活动。进一步拓宽财源渠道，加强财政预算收支管理，加大财政投资审核审计力度，确保财政平稳运行。 四、统筹城乡发展，打造宜居美丽新朝阳

　　加强生态文明建设。 启动大规模国土绿化行动，以创建国家森林城市为抓手，抓好重点区域绿化，完成造林绿化面积60万亩、水土流失综合治理面积70万亩。加大生态系统保护力度，做好生态保护红线、永久基本农田、城镇开发边界控制线划定工作。实施大气、水、土壤综合治理，抓好环城生态绿廊建设、农村环境综合治理、矿山矿业综合整治，进一步改善生产生活条件。持续推进生态环境突出问题整改，扎实做好领导干部自然资源资产离任审计工作。严把项目准入关，绝不以牺牲环境为代价换取暂时的发展。 提高城乡规划建设管理水平。坚持规划引领，做好重点区域、重点项目规划编制工作。统筹城乡发展，协调推进中心城区、县城、新市镇、特色镇、城市社区、宜居乡村建设。加强城市精细化管理，抓好小街小巷综合治理，下力量解决马路市场、交通拥堵、小区物业、沿街占路经营和烧烤烧纸等问题。规范垃圾分类、渣土排放，强化城市道路湿式无尘清扫保洁。继续推进农村饮水安全巩固提升工程，做好农村垃圾处理等工作，建成一批示范乡镇、示范村。

　　抓好基础设施建设。推进中心城区和县城主要道路提质改造，完成朝阳城区北部集中供热改造主体工程，实现管道天然气全线贯通。抓好“四好农村路”建设，持续改善农村交通基础设施条件。加快推进京沈客专、喀赤客专、朝凌客专及锦承、叶赤铁路扩能改造、文化路公铁立交桥等重大工程，实现京沈客专沈阳至朝阳段正式通车，做好锦承线叶柏寿至凌源电气化改造工程前期工作。继续推进迎接高铁时代工作，充分放大高铁效应，掌握发展主动权。

　　五、着力保障和改善民生，进一步创新社会治理 扎实推进脱贫攻坚。完善大扶贫格局，用足用好深度贫困县相关政策措施，深化实化各项扶贫举措，带动解决朝阳整体贫困问题。注重扶贫同扶志、扶智相结合，形成长期稳定脱贫机制，保证现有国家扶贫标准下的脱贫质量，稳定实现农村贫困人口不愁吃、不愁穿，保障义务教育、基本医疗和住房安全，做到脱真贫、真脱贫。动员全社会力量，确保完成4个贫困县摘帽、239个贫困村销号、3.99万贫困人口脱贫任务。

　　全力做好民生保障工作。实施积极的就业创业政策，促进适龄人口尤其是高校毕业生、城镇就业困难人员、农民工等特殊群体就业和返乡创业，城镇新增就业1.6万人。扎实推进各类社会保险统筹扩面，积极做好城乡低保、社会救助、优抚安置、危房改造、冬季供暖等工作，进一步健全残疾人、农村留守儿童和妇女、老年人关爱服务体系。

　　促进社会事业协调发展。实施“第三期学前三年行动计划”，新建改扩建城乡幼儿园16所；推动义务教育均衡发展，促进高中教育优质特色发展；加快现代职业教育体系建设；加大师专转型力度，发展本科教育，积极争取省内高校在我市设立校区。提高公共卫生服务水平，深化公立医院综合改革，加快建设社区卫生服务中心、乡镇卫生院和村卫生室。进一步建立完善公共文化服务体系，繁荣发展文化事业。加强公民道德和精神文明建设，培育和践行社会主义核心价值观。强化计生管理服务，提升计生家庭发展能力。实施全民健身计划，参加十三届省运会并力争再创佳绩。全面贯彻党的宗教方针，深化民族团结进步教育，支持民族地区加快发展。开展全国第四次经济普查和第三次土地调查。做好双拥共建工作，推动军民深度融合发展。

　　提升社会治理水平。夯实基层基础工作，提高社会治理社会化、法治化、智能化、专业化水平，打造共建共治共享的社会治理格局。深入开展“七五”普法，推进法律服务惠民行动。加强应急管理体系建设，提高防灾减灾救灾能力。树立安全发展理念，强化隐患排查治理，严控一般事故、遏制较大事故、坚决杜绝重特大事故。有效控制信访增量，积极化解信访存量，切实解决群众合理诉求。进一步强化食品药品安全监管，积极推进食品安全示范城市创建活动，努力让人民群众饮食用药安全放心。编制全方位立体化公共安全网，强化公共视频监控系统建设和社会信息资源整合，依法打击各类违法犯罪活动。〖HTK〗〖CX3〗

　　六、加强政府自身建设，努力建设人民满意的服务型政府

　　面对艰巨复杂的发展任务，新一届政府要强化担当，主动作为，全面提升施政能力和服务水平，全面提高公信力和执行力，切实担负起振兴发展的神圣使命。

　　坚持正确的政治方向。认真贯彻党的十九大精神，落实全面从严治党要求，推进“两学一做”学习教育常态化制度化，牢固树立“四个意识”“四个自信”。持之以恒落实中央“八项规定”精神，坚持不懈纠正“四风”，深入开展党风廉政建设和反腐败斗争。切实履行“一岗双责”，把抓业务同抓党建、抓队伍、抓廉政紧密结合起来，把管事同管思想、管作风贯通起来，进一步营造良好的政治生态环境，以抓好党建的成效促进政府工作提升。

　　扎实推进依法行政。政府公职人员要心中高悬法律明镜，切实把对法治的尊崇、对法律的敬畏转化为思维方式和行为方式。认真执行人大决议决定，高度重视政协协商议政成果，自觉接受人大及其常委会法律监督、工作监督和政协民主监督，主动接受社会监督和舆论监督。提高政府立法水平，促进立法和改革决策衔接。进一步规范行政决策合法性审查，确保决策科学化、民主化、制度化。推进政务公开，增强政府工作透明度。

　　进一步解放思想。破除传统观念束缚，大胆创新，勇于突破，把解放思想落实到解决问题上、落实到工作成效上。增强机遇意识和紧迫意识，努力快人一拍、先人一步，走在前列，赢得主动。加强学习，提高本领，特别要提升创造性开展工作的能力。对标先进地区，站在更高层次、以更广视野对政府工作进行新的谋划，形成更好的发展格局和态势。

　　切实加强作风建设。坚持目标导向和问题导向，树立服务意识，进一步增强执行力，提高工作效率，以钉钉子精神狠抓落实。强化责任担当，扎实做好打基础、利长远的工作，绝不急功近利搞“形象工程”“政绩工程”。加强效能监察和实绩考核，坚决克服懒政、怠政，对不担当、不作为行为严肃追责问责。建立健全容错纠错机制，为干事者撑腰，替担当者负责。 各位代表，朝阳发展的蓝图已经绘就，奋斗的目标已经明确。我们要高举习近平新时代中国特色社会主义思想伟大旗帜，全面贯彻党的十九大精神，在省委、省政府和市委的坚强领导下，进一步解放思想，攻坚克难，开拓进取，真抓实干，为决胜全面建成小康社会、创造朝阳人民更加美好的生活而努力奋斗！
